# Applying Intervention Mapping to Improve the Applicability of Precious Memories, an Intervention for Depressive Symptoms in Nursing Home Residents

**DOI:** 10.3390/ijerph16245163

**Published:** 2019-12-17

**Authors:** Iris van Venrooij, Jan Spijker, Gerben J. Westerhof, Ruslan Leontjevas, Debby L. Gerritsen

**Affiliations:** 1 Department of Primary and Community Care, Radboud Institute for Health Sciences, Radboud University Medical Center, 6500HB Nijmegen, The Netherlands; iris.vanvenrooij@radboudumc.nl (I.v.V.); roeslan.leontjevas@ou.nl (R.L.); 2 Radboudumc Alzheimer Center, 6500HB Nijmegen, The Netherlands; 3 Behavioural Science Institute, Radboud University, 6500 HE Nijmegen, The Netherlands; j.spijker@propersona.nl; 4 Pro Persona in Mental Health Care, Depression Expertise Centre, 6525DX Nijmegen, The Netherlands; 5 Department of Psychiatry, Radboud University Medical Center, 6500HB Nijmegen, The Netherlands; 6 Department of Psychology, Health, and Technology, Center for Ehealth and Well-Being Research, University of Twente, 7522 NB Enschede, The Netherlands; g.j.westerhof@utwente.nl; 7 Faculty of Psychology and Educational Sciences, Open University of the Netherlands, 6401 DL Heerlen, The Netherlands

**Keywords:** depression, nursing home, psychosocial intervention, applicability, implementation, life review therapy, intervention mapping, process evaluation, maintenance

## Abstract

Precious memories (PM) is a life review intervention for depression in older adults with no to mild cognitive decline that has been implemented in multiple nursing homes (NHs) in the Netherlands. Previous research suggested its relevance but questioned its applicability. Therefore, this research aimed to (1) investigate the applicability of PM, and (2) increase its applicability, if necessary. Intervention mapping (IM) was used to achieve these goals: process evaluation through semi-structured interviews with psychologists (*n* = 11) and clients (*n* = 2) to identify potential improvements for PM and to set an improvement goal (IM-step 1); three focus groups with stakeholders (*n* = 20) to specify behaviors necessary to reach the improvement goal (IM-step 2); and selection of behavior change techniques and applications to facilitate attainment of these behaviors (IM-step 3). Results showed that psychologists perceived a high drop-out rate, which was partly due to PM being provided to clients that did not belong to the target group. Although PM was generally considered relevant, psychologists articulated its longer-term effects should be improved. To improve PM’s applicability, concrete maintenance strategies were developed aiming to maintain clients’ well-being by stimulating positive contact with others. Future research must pilot, implement and evaluate these strategies.

## 1. Introduction

Depression is a common problem in nursing home (NH) residents. An extensive Dutch study [1] has demonstrated that approximately 41% of residents in NH units providing predominantly somatic care, and 52% of residents in dementia special care units showed signs of depression, with 17% and 23% respectively showing signs of severe depression. Depression is associated with adverse consequences in older adults, including poor general health, reduced quality of life and physical and mental functioning, and increased use of medication [2,3]. This illustrates the need for adequate treatment of depression in NH residents.

One treatment for depression in older adults is life review therapy, in which individuals reflect on their life experiences with a therapist [4]. Life review therapy aims to change negative reminiscence styles [5], which have been associated with depressive symptomatology [6]. A meta-analysis [7] concluded that life review therapy is effective in decreasing depressive symptoms at short-term follow-up, and that it might be a promising treatment for depression in older adults in primary care.

Besides negative reminiscence styles, depression has also been related to decreased memory-specificity; older adults with depressive symptoms retrieve fewer specific and more overgeneral memories than older adults without depression [8,9,10]. Additionally, individuals with depression seem to experience most difficulty in retrieving specific positive memories [11]. Specific memories are defined as memories tied to a specific time and place, whereas overgeneral memories only specify a life period and a general event [12]. Several studies have demonstrated that memory-specificity can be trained [13,14,15], and a meta-analysis [16] has concluded that increases in memory-specificity are associated with small to moderate improvements in depressive symptoms in (older) adults.

Precious memories (PM) is an intervention that combines life review therapy with training memory-specificity for positive events. PM is considered a life review therapy [17], because it systematically works through multiple life periods. Several studies have shown that PM can have significant beneficial effects on depressive symptoms [13,15,18,19], life satisfaction [13,15], and hopelessness [15] in older adults. Although PM was developed for use in the general older population, it has also been implemented in several NHs in the Netherlands as part of Act in Case of Depression—a comprehensive program for the detection and treatment of depression in NH residents [1].

However, a process evaluation of this program in a Dutch trial [20] showed that although PM was received positively by health care professionals, it was not often applied in daily practice. Therefore, it was suggested that its applicability—PM’s usefulness and relevance—in the NH setting should be further investigated and that improvements might be needed. Furthermore, an effect study [19] of PM for depressive symptoms in NH residents showed that reductions in depressive symptoms and increases in memory-specificity were not maintained at eight-month follow-up. These results might also be explained by PM’s potential limited applicability to the NH population. Therefore, the aims of this study are (1) to further investigate the applicability of PM in NH practice, and, subsequently, (2) to develop strategies to increase its applicability in this setting.

## 2. Materials and Methods

### 2.1. Design

The two aims of this study were addressed based on available evidence and theory, which is considered best practice by the Medical Research Council [21]. We chose intervention mapping (IM) [22,23] to systematically increase the applicability of PM. Although IM is predominantly used as a method to systematically develop new interventions, it might also help to adapt existing interventions to new settings and populations [24]. IM aims to provide guidelines for effective decision-making during the development and adaptation of an intervention, integrating theory, empirical findings, and information from the target population [24]. Six steps are described to develop and evaluate an intervention: (1) identification of potential improvements and setting an improvement goal (“needs assessment”); (2) defining behaviors and their determinants, needed to reach the improvement goal (“matrices of change objectives”); (3) selecting behavior change techniques and ways to apply them (“theory-based methods and practical applications”); (4) “program production”; (5) “adoption and implementation”; and (6) “evaluation planning” [24] (pp. 20–24). IM-step 1 was used to address the first aim, and IM-steps 2 and 3 to address the second aim of this study.

### 2.2. Intervention

PM has been applied in multiple NHs in the Netherlands and is considered relevant in NH residents with mild to moderate depressive symptoms having no to mild cognitive decline [19,25]. PM consists of five sessions, commonly provided by a trained psychologist. The first is an introductory session in which the psychologist (1) evaluates to what extent a client can be trained to retrieve specific, positive memories, (2) explains PM and sets therapeutic goals, and (3) identifies life phases that are appropriate for retrieving positive memories. This is followed by three sessions focusing on childhood, adolescence and adulthood, and a concluding session [25].

### 2.3. Procedure According to Intervention Mapping

**Figure 1:**
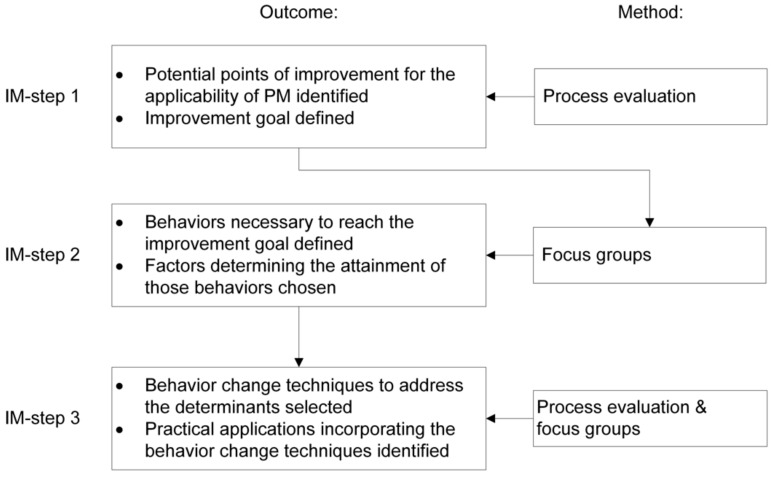
Overview of intervention mapping (IM)-steps, outcomes and methods to reach those outcomes.

Because we used IM to address the goals of this study, methods (and results) are described following those steps. An overview of the outcomes of each IM-step and methods to reach those outcomes can be found in Figure 1.

#### 2.3.1. IM-Step 1: Identification of Potential Improvements and Setting an Improvement Goal

Since previous research concluded that PM’s applicability to the NH setting might be problematic [20], we investigated factors contributing to PM’s applicability that could be improved. Because PM has already been applied in the NH setting, we had the opportunity to identify potential improvements based on a process evaluation of the current situation as experienced by stakeholders and to collect information on potential improvements. A process evaluation can provide insight into factors that diminish or increase the effects of an intervention [21], barriers and facilitators to implementation [20] and acceptability and feasibility of an intervention [26], and might therefore provide suggestions on how to optimize an intervention and its implementation strategies [21]. To this end, the framework of Leontjevas et al. [20] was used, which has successfully been applied in NH studies [27,28,29]. This framework distinguishes between first and second-order processes. First-order processes reflect (1) reach and sampling quality, describing selection procedures for the intervention and completion rates, and (2) intervention quality—which is defined as the extent to which components of PM are conducted according to the initial protocols and the extent to which PM is evaluated as relevant, clear, and feasible. Second-order process data provide insight into implementation strategies, and barriers and facilitators to implementation. Based on the outcomes of the process evaluation, the researchers (I.v.V., J.S., and D.L.G.) identified potential points of improvement. After choosing the most relevant one to focus on in this study, an improvement goal for PM was defined (Figure 1).

For data collection, we planned to conduct semi-structured interviews with a minimum of 10 psychologists who had followed the PM training and with 10 clients, and to stop conducting interviews if no new themes would arise after three consecutive interviews [30]. For psychologists, purposive sampling was used to increase data saturation [30], based on the year in which psychologists followed a PM training and the type of clients they worked with. Prior to the interview, psychologists completed an online questionnaire, covering demographic and occupational information, reach, relevance, feasibility, and barriers and facilitators (results are available in Table S1). The scores on the online questionnaire of a psychologist were used to further specify the content of the interview with that psychologist. Clients who had received PM were invited for semi-structured interviews through the contacted psychologists. The interviews were conducted by one of the researchers (I.v.V.).

We operationalized the elements of the process evaluation framework into topic guides based on other published process evaluations [27,29]. All interviews were transcribed and coded by the first author following a content analysis approach [31], meaning that codes were assigned inductively, as well as deductively, based on the process evaluation framework. After initial codes were assigned, categories were created. Subsequently, a tree diagram was developed depicting the relationships between codes and their categories, as agreed upon by the researchers (I.v.V., J.S. and D.L.G.). The qualitative data were manually coded in ATLAS.ti (version 7.1.5.).

#### 2.3.2. IM-Step 2: Defining Behaviors and Their Determinants, Needed to Reach the Improvement Goals

We conducted three focus groups with psychologists and nursing staff who had experience with PM to define behaviors necessary to achieve the improvement goal of PM and factors determining the attainment of those behaviors (Figure 1).

In Focus Group 1, participants generated ideas on ways to address the most relevant point of improvement of PM. In Focus Group 2, generation was continued after which consensus was obtained on the best strategy to address this point using the nominal group technique [32]. Prior to this focus group, participants were asked to consider how to practically address the point of improvement. During the focus group, participants, first individually, wrote down ideas on how to address the improvement goal (silent generation). Secondly, participants named their ideas until all ideas were explained and discussed (round robin). Thirdly, the participants prioritized the ideas based on potential effects and feasibility (voting). Participants could also provide new strategies. In Focus Group 3, the strategy identified as most relevant in Focus Group 2 was discussed further to obtain specific information on its implementation in practice.

Focus group participants were recruited based on convenience sampling. The focus groups were conducted using topic guides. Using tape-based analysis [33], the content of the focus groups was arranged according to topic and summarized.

#### 2.3.3. IM-Step 3: Selecting Behavior Change Techniques and Ways to Apply Them

To address the determinants of the behaviors needing change, behavior change techniques and practical applications were selected. Furthermore, specific conditions under which behavior change techniques are effective were specified (Figure 1).

Together with a clinical psychologist who provides training in PM, the researchers (I.v.V., J.S., and D.L.G.) selected the behavior change techniques and conditions under which these are effective based on behavior change theories [23,34,35], and chose applications based on the outcomes of the process evaluation and focus groups.

### 2.4. Ethical Approval

The local Medical Ethics Review Committee (CMO Region Arnhem-Nijmegen) reviewed the study and declared the study not burdensome to participants (number 2017–3854)**.** The study was conducted in accordance with the Declaration of Helsinki and the applicable Dutch legislation. All participants signed an informed consent form before participation in the study.

## 3. Results

### 3.1. Participants

Face-to-face interviews were conducted with Dutch NH residents with depressive symptoms (Table 1). A third client was interviewed; however, because she seemed anxious, her data were not used. All interviews with psychologists (Table 2) were conducted in Dutch and by telephone, except for one interview which was conducted face-to-face because of participant preference. Characteristics of focus group participants are included in Table 2.

### 3.2. IM-Step 1: Identification of Potential Improvements and Setting an Improvement Goal

#### 3.2.1. First-Order Process Evaluation

Reach: psychologists believed they proposed PM to less than half of the estimated indicated population, mainly because of time constraints for individual treatments, especially for clients with cognitive impairments.

Completion: psychologists reported multiple instances in which they applied PM to clients with moderate or severe cognitive impairments, which was an exclusion criterion for using PM. Furthermore, psychologists estimated that approximately two-thirds of the clients dropped out of therapy, which in their opinion was mainly due to clients’ limited cognitive abilities, such as a limited attention span and an inability to (be trained to) retrieve specific, positive memories.

Intervention quality: PM was generally regarded as relevant by psychologists and as pleasant by clients, mainly because of its positive focus. Furthermore, some psychologists perceived positive effects on mood, even for clients with moderate or severe cognitive impairments, who were unable to remember the session’s content and could not be trained to retrieve specific, positive memories. However, it was also reported that some clients with more severe cognitive impairments were still able to retrieve specific, positive memories, especially from childhood and adolescence. Nearly all interviewed psychologists would recommend the use of PM to other psychologists.

Although psychologists and clients perceived positive effects on mood during and in between sessions, they questioned longer-term effects of PM, due to the restricted trainability of clients. Both expressed that maintenance of the effects of PM should be improved and should be the improvement goal for PM. They reported that clients might only retrieve general memories directly after PM and might, therefore, need the help of others in maintenance. Accordingly, clients also expressed difficulties in retrieving memories by themselves. Incorporating the retrieved memories in mediative interventions (i.e., interventions delivered through others) was mentioned as a strategy for maintenance. Thus, although the interviewed psychologists considered providing PM a task for psychologists—because particular communication skills are necessary to reach specific, positive memories—the involvement of others was suggested for maintaining the effects.

#### 3.2.2. Second-Order Process Evaluation

Barriers and facilitators: the PM training and protocol were reported as facilitators to implementation, whereas mentioned barriers were a lack of time and continuity because of changes within the organization and team, a medically oriented organizational culture, and a relatively low importance of PM within the organization. Furthermore, the attitude of close colleagues, such as other psychologists, could be a barrier if those had different priorities than implementing PM, or limited experience with PM.

#### 3.2.3. Setting the Improvement Goal of PM

The process evaluation showed that it was indeed necessary to improve the applicability of PM to the NH setting and identified that (1) the reach of PM, (2) its low completion rates, and (3) its perceived low level of maintenance of effects must be improved. The first point primarily requires structural changes in the NH: allocating more time to individual treatments, which requires the involvement of the management of NHs and, potentially, health care insurance providers, which was therefore not addressed in this study. Regarding the second point of improvement, low completion rates may also stem from PM having been started in residents with more severe cognitive impairments whereas ‘having no to mild cognitive impairment’ was an inclusion criterion for PM. Decreased trainability of these clients was namely the most reported reason for dropout. The third point, maintenance of effects, emerged as the primary point of improvement from the interviews. Accompanied by the finding that reductions in depressive symptoms were not maintained at eight-month follow-up after applying PM to NH residents [19], we chose to set the improvement goal of PM to increase maintenance of the PM’s effects. A model illustrating which behaviors and determinants contribute to the perceived low level of maintenance of PM’s effects (IM: logic model of the problem) is available in Figure S1.

### 3.3. IM-Step 2: Defining Behaviors and Their Determinants, Needed to Reach the Improvement Goal

#### 3.3.1. Focus Groups

In Focus Group 1, participants generated ideas on ways to achieve maintenance of PM’s effects. Nursing staff and informal caregivers were regarded as individuals who could be practically involved in executing the maintenance strategies. The strategy identified for the transition between PM and maintenance was the psychologist discussing maintenance strategies with the client and informal caregiver at the end of PM, in regular care, or during family meetings.

In the Focus Group 2, participants continued the idea generation of Focus Group 1 and obtained consensus on the best idea. During the idea generation, participants discussed several issues concerning the goal and execution of the maintenance strategies. Firstly, they questioned whether the goal of maintenance should be to train memory-specificity; participants noted that this is not feasible for individuals with prominent cognitive impairments, which they regarded as a relevant target group for PM. Secondly, the positive nature of the conversations in the PM sessions, which results from retrieving positive memories, was thought to be beneficial to clients. Accordingly, participants found the goal of the maintenance strategies should be to provide clients with continued positive contact, allowing persons other than the psychologist, such as nursing staff, informal caregivers, family members and volunteers (henceforth referred to as ‘supporters’) to be involved in maintenance. The following aspects were considered prerequisites for maintenance:

- Psychologists should be in charge of PM, but maintenance could be coordinated by a nurse or family member;
- A maintenance plan should be included in clients’ dossiers and should include distinct maintenance advice for nursing staff;
- The PM protocol should include a checklist on what information to include in clients’ dossiers;
- The retrieved memories must be preserved after PM;
- Clients must be involved in the decision with whom to share which memories;
- Individuals involved in maintenance should have knowledge about the general principles of PM.

Due to limited time, it was not possible to group and vote on the generated ideas during the focus group. Therefore, after the focus group, the ideas were grouped by I.v.V. and D.L.G. Focus group participants were then asked by email to prioritize the strategies from least to most helpful, based on potential effects and feasibility. Table 3 shows the results.

Mediative interventions were identified as the best strategy for providing maintenance. Additionally, since creating a memory book or box (perceived as the second-best strategy) could also be considered a mediative intervention, this was also included in the discussion of Focus Group 3.

Participants of Focus Group 3 were asked how mediative interventions could be implemented in practice. Participants discussed that supporters could be involved in maintenance strategies aimed at providing positive contact with the client. They would need to be instructed and provided with ideas on topics to ask questions about, cues that helped to retrieve positive memories during PM, and with information about appropriate life periods to discuss.

Lastly, the transfer between the psychologist and those involved in maintenance was discussed. The maintenance plan should include information on which life periods the client enjoyed talking about and the positive memories the client retrieved. Furthermore, the psychologist should appoint a coordinator to evaluate maintenance, such as a family member or nurse.

#### 3.3.2. Defining Behaviors and Their Determinants

As described, the goal of the maintenance strategies of PM was set to maintain clients’ well-being by strengthening their positive contact with others. To address this goal, supporters help clients to retrieve positive memories, which provides clients with positive contact. Because the interview and focus group results showed that asking for specific, positive memories requires particular communication skills especially in a target group with cognitive impairments, we decided the most feasible strategy would be for others to support the retrieval of positive memories without explicitly focusing on the memories being specific. From the focus groups it followed that supporters need concrete guidelines on what topics to ask questions about. Therefore, they could create so-called memory products, such as life books, photo books/frames, and tool-boxes with objects, which connect to specific positive memories that came up during the PM sessions, facilitating the retrieval of positive memories.

Based on the interview and focus group results, we identified behaviors to reach the improvement goal. The following behaviors were defined, which describe how the maintenance strategies are organized:

1. After each PM session, with permission from the client, the psychologist reports the client’s retrieved positive memories in the client’s dossier;
2. After the five PM sessions, the psychologist and main nurse meet and develop a plan to improve positive contact with the client. They decide who is going to make what kind of memory product, who will help the client retrieve memories with the help of the product and when;
3. The psychologist and the main nurse together integrate helping the client retrieve memories as an activity in the pleasant activities plan of the client;
4. The psychologist instructs and practices the retrieval of positive memories with the supporter;
5. The supporter helps the client to retrieve memories at the—during the meeting decided—times with the help of the memory product;
6. The psychologist and main nurse evaluate (and adjust) the pleasant activities plan of the client as described in the Act in Case of Depression protocol;
7. The psychologist and main nurse implement PM (specifically the steps specified under 1–6).

Additionally, we chose attitudes, knowledge, self-efficacy, and outcome expectations as determinants of the behaviors needed to reach the improvement goal. The first column of Table 4 illustrates how these determinants can facilitate the attainment of one of the behaviors that must be performed to attain the improvement goal. A complete table of behaviors and their determinants (IM: Matrix of change objectives) can be found in Table S2.

### 3.4. IM-step 3: Selecting Behavior Change Techniques and Ways to Apply Them

**Figure 2:**
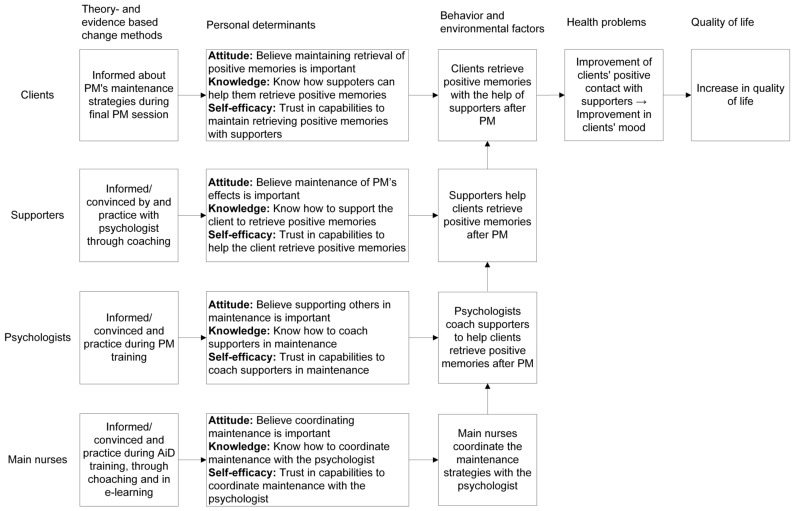
Logic model of change.

To address the determinants of the behaviors needing change, we selected behavior change techniques [23,34,35], conditions under which these techniques are effective and identified ways to deliver those techniques (applications) based on the process evaluation and focus group results. To ensure a fit between the person involved in the maintenance strategies and the applications, we chose different applications for individuals with different roles: the already existing PM training for psychologists, the Act in Case of Depression training and an e-learning for nursing staff (www.doenbijdepressie.nl); and newly developed coaching by the psychologist for nursing staff and supporters. Including the maintenance strategies in the existing training programs would be the most feasible and provides psychologists, nursing staff and supporters with the opportunity to practice the learned techniques and to receive feedback from the trainer. Through the newly developed coaching, the psychologists—who are used to providing psycho-education and coaching to nursing staff—can instruct the nursing staff and supporters on the maintenance strategies and practice these with them. The second, third and fourth column of Table 4 illustrate how and which behavior change techniques were chosen to address the determinants of each of the identified behaviors needed to reach the improvement goal, and consequently, how those behavior-change techniques should be applied. A complete table showing this for all identified behaviors can be found in Table S3. An overview of how the maintenance strategies are designed to increase quality of life (IM: logic model of change) is shown in Figure 2.

## 4. Discussion

This study’s first aim was to investigate the applicability of PM in NH practice. The process evaluation showed that both the reach and completion rates of PM were low. Furthermore, PM was provided to clients with moderate or severe cognitive impairments, contrary to the inclusion criteria. Although PM was generally perceived as relevant and applicable, also in residents with cognitive impairments, its longer-term effects were questioned because of assumed limited trainability of clients, while maintaining PM effects was considered a primary improvement goal. Mentioned barriers to implementation were a lack of time and staff continuity, a medically oriented organizational culture, and a relatively low organizational priority of PM; facilitators were the PM training and protocol.

The second aim was to develop strategies to increase the applicability of PM in the NH setting. The strategies did not focus on PM itself, but regarded increasing maintenance of PM’s effects. Using focus groups, the goal for maintenance was defined: improving clients’ well-being by providing them with positive contact with others through helping them retrieve positive memories after PM ends. Subsequently, based on identified prerequisites for maintenance, maintenance strategies, including the formation of a maintenance plan, were suggested.

An important issue was that psychologists generally did not think their target population was capable of training memory-specificity, part of the supposed working mechanism of PM [13,15,18,19], because of cognitive impairments. In the original PM protocol, clients were assumed to continue retrieving specific, positive memories by themselves, after the intervention had ended. However, for clients with limited trainability, it is more difficult to retrieve specific, positive memories by themselves. Nonetheless, psychologists did perceive positive effects on clients’ mood, which they hypothesized might be caused by the pleasant interactions clients have during sessions, which arise from the retrieval of general positive memories. Accordingly, we incorporated the positive contact with others through retrieving positive memories in the goal of maintenance strategies. A recent systematic review on the effects of life story books [36] found that these types of reminiscence activities can improve autobiographical memory, depression, mood, and quality of life in persons with dementia. Because the maintenance strategies of PM, similar to life story books, can support a person in retrieving positive memories, they might produce similar positive effects by providing clients with reminiscence.

Furthermore, maintenance strategies may account for decreased trainability of clients and, in this way, even increase the applicability of PM to clients with moderate or severe cognitive impairments and thereby also reduce drop out. However, further investigation should examine the applicability of specific aspects of PM to clients with more severe cognitive impairments, such as the questions psychologists use to probe for specific memories, the extent to which clients may deviate from life periods and the extent to which retrieved memories must be specific. Yet, PM was only delivered to less than half of the estimated indicated population because of limited time for individual treatments. Indeed, other NH studies also identified time restrictions as implementation barriers, e.g., [14,21]. This finding illustrates the need to improve the implementation of individual client therapies in the NH setting in general.

This study has a number of strengths. Firstly, the IM protocol was used to systematically evaluate and provide strategies for the improvement of PM. This provided transparency about the evaluation and improvement of PM creating important insights into workable maintenance strategies [24], especially since we integrated an extensive process evaluation in the first IM step. Furthermore, the involvement of the intervention’s stakeholders in the development process, also embedded in IM, helped us to incorporate practically relevant issues [37] and is expected to increase the efficacy of the intervention [38].

Some limitations must also be acknowledged. Firstly, we aimed to use specific criteria for data saturation [30], but, because of time constraints, evaluating saturation after 10 interviews was not feasible. Nonetheless, Francis et al. [30] demonstrated that the use of purposive sampling could increase data saturation to between 86% to 92% after six interviews, suggesting that conducting 11 interviews with purposely sampled psychologists might have contributed to a satisfactory level of data saturation. Furthermore, we could only use two interviews with clients, because it was not feasible to recruit clients without cognitive impairments who had been provided with PM relatively recently.

We originally planned to investigate the implementation strategies used by psychologists to implement the intervention. However, as psychologists in the first three interviews were unable to provide any information regarding these strategies, this topic was removed from the topic list. It is, however, important that individuals implementing an intervention know how to incorporate it into their tasks [39]. To address the found lack of knowledge about implementation strategies, we developed implementation strategies for the improvement goal.

Lastly, we aimed to influence the behavior of individuals directly involved in the maintenance strategies of PM. However, as the found barriers illustrate, when care organizations implement PM and its maintenance strategies, support at higher organizational levels must be obtained [39].

## 5. Conclusions

Individuals who have recovered from a depressive episode are at an increased risk of having another depressive episode in the future [40]. This illustrates the importance of investigating how the effects of psychotherapeutic interventions can be maintained. This research has contributed to this question by suggesting concrete strategies to maintain the effects of a specific intervention for depression in NH residents. In future research, the maintenance strategies of PM must be piloted, implemented, and evaluated on effects and processes (corresponding with IM-steps 4, 5 and 6, respectively). Furthermore, by strengthening PM, such a study may yield an alternative to mediative therapies in NHs, especially for residents with more severe cognitive impairments, thereby acknowledging that NH residents might still be able to receive individual therapies for depression.

## Figures and Tables

**Table 1:** Characteristics of client interviews.

Characteristic	Client 1	Client 2
Age	63	95
Sex	Male	Female
Length of nursing home (NH) stay (years)	2.5	1
Length interview (minutes)	44	29
Months between end of previous memories (PM) and study	3	4

**Table 2:** Characteristics of interviews with psychologists and focus group participants.

Participant Group	*n* (Female)	Mean Age (Range)	Profession	Work Experience in Elderly Care in Years (Range)	Years Between PM Training and Study	Type of Clients Participants Worked With	Length Interviews and Focus Groups in Minutes
Interviews with psychologists	11 (11)	40.18 (29–54)	Psychologist (*n* = 11)	10.09 (5–18)	1 (*n* = 8), 2 (*n* = 1), 4 (*n* = 2)	Somatic (*n* = 5), cognitive (*n* = 3), both (*n* = 2), clients from primary care (*n* = 1)	*M* = 69.91 (13–99)
Focus Group 1	10 (9)	35.30 (25–56)	Psychologist (*n* = 8), clinical neuropsychologist (*n* = 1), psychological assistant (*n* = 1)	8.70 (0.5–24)	0.5 (*n* = 8), 4.5 (*n* = 1), 10 (*n* = 1)	Cognitive (*n* = 5), somatic (*n* = 2), both (*n* = 2), unknown (*n* = 1)	26
Focus Group 2	6 (6)	48.50 (27–60)	Psychologist (*n* = 4), nurse scientist (*n* = 1), nurse (*n* = 1)	16.50 (4–28)	4 (*n* = 2), 1 (*n* = 1), not trained (*n* = 3)	Both (*n* = 3), cognitive (*n* = 1)	106
Focus Group 3	4 (4)	52.25 (41–61)	Psychologist (*n* = 4)	17.13 (5.5–25)	8 (*n* = 3), 1 (*n* = 1)	Somatic (*n* = 2), both (*n* = 2)	49

Cognitive = clients with primarily cognitive impairments; somatic = clients with primarily somatic problems; both = clients with both cognitive impairments and somatic problems.

**Table 3:** Prioritized options for maintenance strategies, from most (1) to least (7) helpful.

Priority	Options for Maintenance Strategies
1	Mediative interventions: (Temporarily) including retrieved memories in mediative interventions by (1) adapting stimuli in the environment of the client to the retrieved memories (e.g., memory walls, scents, and music), (2) having supporters help clients retrieve precious memories, or (3) providing the client with activities based on the retrieved memories.
2	Memory book or box: The memories retrieved during PM are processed into a memory book or box after PM.
3	Follow-up session: Providing a follow-up session after the end of PM to assess whether clients need more guidance to maintain the retrieval of specific, positive memories.
4	Mini PM-sessions: Using mini PM-sessions (i.e., booster sessions) after the end of PM to maintain the skill to retrieve specific, positive memories.
5	Diary/letter: Potentially with help, clients write up their retrieved memories in a diary or letter to themselves.
6	Embedding PM in other therapies: Including PM in other (psychological) therapies.
7	Group treatment: Clients who received PM can afterwards participate in a group treatment to maintain the skill to retrieve specific, positive memories.

**Table 4:** Explication of determinants, behavior-change techniques, applications and conditions for one of the behaviors * needed to reach the improvement goal.

Determinants of the Behavior *	Behavior Change Techniques	Application	Explanation (Conditions under Which Behavior Change Techniques are Effective in Bold)
Attitude: The psychologist believes it is important to report the positive memories.	Arguments [23] to convince the psychologist of the importance of reporting the positive memories.	PM training	Psychologists are informed that reporting the positive memories is necessary to create memory-products (**new information**). (Outcome expectation 1)
Knowledge (a) The psychologist knows why it is important to report the positive memories.	Providing information [23,34] about the expected outcome of this behavior.	PM training	The information is provided by the PM trainer.
(b) The psychologist knows how to report the positive memories.	Individualized [23] instruction [34,35] by providing a model [23,34,35] of the desired outcome.	PM training	Psychologists are instructed by the PM trainer (**appropriate model**) on how to report the positive memories and are shown an example of how to report them. Opportunity to ask questions (**responding to needs**).
Self-efficacy: The psychologist feels capable to report the positive memories.	Psychologists are verbally persuaded [23,34,35] about their capabilities. Guided practice [23,34] with feedback on performance [34,35].	PM training	Psychologists are persuaded by the PM trainer (**credible source**) why they are capable to report the memories. They practice and receive feedback on performance (**specific and individual**) from the PM trainer (**experienced person**) in the second PM training session.

* One of the behaviors needed to reach the improvement goal is “After each PM session, with permission from the client, the psychologist reports the client’s retrieved positive memories in the client’s dossier”.

## Data Availability

The data that support the findings of this study are available from the corresponding author on request.

## Supplementary Materials

The following are available online at https://www.mdpi.com/1660-4601/16/24/5163/s1, Table S1: online questionnaire, Table S2: matrix of change objectives, Table S3: selected theory-based methods and applications addressing the determinants of behavior change, Figure S1: logic model of the problem.
