# Supplementary material for: Applying Intervention Mapping to Improve the Applicability of Precious Memories, an Intervention for Depressive Symptoms in Nursing Home Residents
Source: Int J Environ Res Public Health. 2019 Dec 17;16(24):5163. doi: 10.3390/ijerph16245163 (PMC6950488; doi:10.3390/ijerph16245163)
Supplement: Supplementary file 1 [file ijerph-16-05163-s001.zip › Supplemental final/Table S2 Matrix of change objectives.pdf]

Table S2. Matrix of change objectives

Necessary behavior (Performance objective; PO)	Determinants				
	Attitude (A)	Knowledge (K)		Self-efficacy (SE)	Outcome expectation (OE)
PO1: After each PM session, with permission from the client, the psychologist reports the client's retrieved positive memories in the client's dossier.	A1: The psychologist believes it is important to report the positive memories.	K1a: The psychologist knows why it is important to report the positive memories.	K1b: The psychologist knows how to report the positive memories.	SE1: The psychologist feels capable to report the positive memories.	OE1: The psychologist expects that reporting the positive memories is necessary to create a memory product.
PO2: After the five PM sessions, the psychologist and main nurse meet and develop a plan to improve positive contact with the client. They decide who is going to make what kind of memory product, who will help the client retrieve memories with the help of the product and when.	A2: The psychologist and main nurse believe it is important to develop a detailed plan to improve positive contact together with the client.	K2a: The psychologist and main nurse know why it is important to develop a detailed plan to improve positive contact with the client.	K2b: The psychologist and main nurse know how to develop a detailed plan to improve positive contact with the client.	SE2: The psychologist and main nurse feel capable to develop a detailed plan to improve positive contact with the client.	OE2: The psychologist and main nurse expect that developing a plan increases application of PM.

PO3: The psychologist and the main nurse together integrate helping the client retrieve memories as an activity in the pleasant activities plan of the client.	A3: The psychologist and main nurse believe it is important to integrate the activity in the pleasant activities plan together.	K3a: The psychologist and main nurse know why it is important to integrate the activity in the pleasant activities plan together.	K3b: The psychologist and main nurse know how to integrate the activity in the pleasant activities plan together.	SE3: The psychologist and main nurse feel capable to integrate the activity in the pleasant activities plan together.	OE3: The psychologist and main nurse expect that integrating the activity in the pleasant activities plan together increases application of PM.
PO4: The psychologist instructs and practices the retrieval of positive memories with the supporter.	A4: The psychologist believes it is important to instruct and practice with the supporter.	K4a: The psychologist knows why it is important to instruct and practice with the supporter.	K4b: The psychologist knows how to instruct and practice with the supporter.	SE4: The psychologist feels capable to instruct and practice with the supporter.	OE4: The psychologist expects that instructing and practicing with the supporter increases the quality of the activity.
PO5: The supporter helps the client to retrieve memories at the—during the meeting decided—times with the help of the memory product.	A5: The supporter believes it is important to help the client retrieve positive memories.	K5a: The supporter knows why it is important to help the client retrieve positive memories.	K5b: The supporter knows how to help the client retrieve positive memories.	SE5: The supporter feels capable to help the client retrieve positive memories.	OE5: The supporter expects that helping the client retrieve positive memories improves contact with the client.
PO6: The psychologist and main nurse evaluate (and	A6: The psychologist and	K6a: The psychologist and	K6b: The psychologist and	SE6: The psychologist and	OE6: The psychologist expects that

adjust) the pleasant activities plan of the client as described in the Act in Case of Depression protocol.	main nurse believe it is important to evaluate (and adjust) the pleasant activities plan together.	main nurse know why it is important to evaluate (and adjust) the pleasant activities plan together.	main nurse know how to evaluate (and adjust) the pleasant activities plan together.	main nurse believe they are capable to evaluate (and adjust) the pleasant activities plan together.	evaluating (and adjusting) the pleasant activities plan improves contact with the client.
PO7: The psychologist and main nurse implement PM (specifically the steps specified under PO1-6).	A7: The psychologist and main nurse believe implementing PM is part of their tasks.	K7a: The psychologist and main nurse know why it is important to implement PM.	K7b: The psychologist and main nurse how to implement PM.	SE7: The psychologist and main nurse feel capable to implement PM.	OE7: The psychologist and main nurse believe implementation is necessary to execute PM properly.
