# Supplementary material for: Applying Intervention Mapping to Improve the Applicability of Precious Memories, an Intervention for Depressive Symptoms in Nursing Home Residents
Source: Int J Environ Res Public Health. 2019 Dec 17;16(24):5163. doi: 10.3390/ijerph16245163 (PMC6950488; doi:10.3390/ijerph16245163)
Supplement: Supplementary file 1 [file ijerph-16-05163-s001.zip › Supplemental final/Table S3 Selected theory-based methods and applications addressing the determinants of behavior change.pdf]

Table S3. Selected theory-based methods and applications addressing the determinants of behavior change

Determinants	Who	Theory-based method(s)	Application(s)	Explanation (parameters)
Attitude (A)				
A1: The psychologist believes it is important to report the positive memories.	P	**Arguments** [1] to convince the psychologist of the importance of reporting the positive memories.	PM training	Psychologists are informed that reporting the positive memories is necessary to create memory-products (**new information**). (OE1)
A2: The psychologist and main nurse believe it is important to develop a detailed plan to improve positive contact together with the client.	P	**Arguments** [1] to convince the psychologist to develop a detailed plan together with the main nurse to improve positive contact with the client.	PM training	Psychologists are informed that developing a detailed plan together with the main nurse increases application of PM (**new information**). (OE2)
	N	**Arguments** [1] to convince the main nurse to develop a detailed plan together with the psychologist to improve positive contact with the client.	*AiD training and e-learning*	Nurses are informed that developing a detailed plan together with the psychologist increases application of PM (**new information**). (OE2)
A3: The psychologist and main nurse believe it is important to integrate the activity in the pleasant activities plan together.	P	**Arguments** [1] to persuade the psychologist of the importance of integrating the activity in the pleasant activities plan together with the main nurse.	PM training	Psychologists are informed that integrating the activity in the pleasant activities plan increases application of PM (**new information**). (OE3)

	N	**Arguments** [1] to persuade the main nurse of the importance of integrating the activity in the pleasant activities plan together with the psychologist.	*AiD training and e-learning*	Nurses are informed that integrating the activity in the pleasant activities plan increases application of PM (**new information**). (OE3)
A4: The psychologist believes it is important to instruct and practice with the supporter.	P	**Arguments** [1] to convince the psychologist of the importance of instructing and practicing with the supporter.	PM training	Psychologists are informed that instructing and practicing with the supporter increases quality and application of PM (**new information**). (OE4)
A5: The supporter believes it is important to help the client retrieve positive memories.	<u>S</u>	**Arguments** [1] to persuade the supporter of the importance of helping the client retrieve positive memories. The supporter **directly experiences** [1] the effect of retrieving positive memories through **active learning** [1].	<u>Coaching by psychologist</u>	Supporters are instructed by the psychologist that retrieving positive memories with the client improves contact (**new information**) and practice multiple questions with the psychologist through role play. Afterwards, supporters are asked how it felt and how it could affect the client (**explaining outcomes**). (OE5)
A6: The psychologist and main nurse believe it is important to evaluate (and adjust) the pleasant activities plan together.	P	**Arguments** [1] to convince the psychologist to evaluate (and adjust) the pleasant activities plan together with the main nurse.	PM training	Psychologists are told that evaluating (and adjusting) the pleasant activities plan improves the quality of the plan and is a requirement to improve client contact (**new information**). (OE6)
	N	**Arguments** [1] to convince the main nurse to evaluate (and adjust) the	*AiD training and e-learning*	Nurses are told that evaluating (and adjusting) the pleasant activities plan

		pleasant activities plan together with the psychologist.		improves the quality of the plan and is required to improve client contact (**new information**). (OE6)
A7: The psychologist and main nurse believe implementing PM is part of their tasks.	P/*N*	**Arguments** [1] to convince the psychologist and main nurse that implementing PM is part of their tasks. **Providing information about approval** [1–3] of the management of the NH.	PM training/*AiD training and e-learning*	Psychologists and nurses are told that implementation is necessary to execute PM properly (**new information**) and that the management of the NH supports this (**positive expectations**). (OE7)
Knowledge (K)				
K1a: The psychologist knows why it is important to report the positive memories.	P	**Providing information** [1,2] about the expected outcome of this behavior.	PM training	The information is provided by the PM trainer.
K1b: The psychologist knows how to report the positive memories.	P	**Individualized** [1] **instruction** [2,3] by providing a **model** [1–3] of the desired outcome.	PM training	Psychologists are instructed by the PM trainer (**appropriate model**) on how to report the positive memories and are shown an example of how to report them. Opportunity to ask questions (**responding to needs**).
K2a: The psychologist and main nurse know why it is important to develop	P	**Providing information** [1,2] about the expected outcome of this behavior.	PM training	The information is provided by the PM trainer.

a detailed plan to improve positive contact with the client.	*N*	**Providing information** [1,2] about the expected outcome of this behavior.	*AiD training and e-learning*	The information is provided by the AiD trainer and in the e-learning.
K2b: The psychologist and main nurse know how to develop a detailed plan to improve positive contact with the client.	P	**Individualized** [1] **instruction** [2,3] on how to construct a plan with the necessary components through **active learning** [1] and **demonstration** [1–3].	PM training	Psychologists are instructed by the PM trainer (**appropriate model**), who demonstrates an example plan. Opportunity to ask questions (**responding to needs**).
	N	**Individualized** [1] **instruction** [2,3] on how to construct a plan with the necessary components through **active learning** [1] and **demonstration** [1–3].	*AiD training and e-learning*	Nurses are instructed by the AiD trainer (**appropriate model**), who demonstrates an example plan. Opportunity to ask questions (**responding to needs**).
K3a: The psychologist and main nurse know why it is important to integrate the activity in the pleasant activities plan together.	P	**Providing information** [1,2] about the expected outcome of this behavior.	PM training	The information is provided by the PM trainer.
	N	**Providing information** [1,2] about the expected outcome of this behavior.	*AiD training and e-learning*	The information is provided by the AiD trainer and in the e-learning.
K3b: The psychologist and main nurse know how to integrate the	P	**Individualized** [1] **instruction** [2,3] and **demonstration** [1–3] of an example plan.	PM training	Demonstration and instruction by the PM trainer (**appropriate model**); opportunity to ask questions (**responding to needs**).

activity in the pleasant activities plan together.	*N*	**Individualized** [1] **instruction** [2,3] and **demonstration** [1–3] of an example plan.	*AiD training*	Demonstration and instruction by the AiD trainer (**appropriate model**); opportunity to ask questions (**responding to needs**).
K4a: The psychologist knows why it is important to instruct and practice with the supporter.	P	**Providing information** [1,2] about the expected outcome of this behavior.	PM training	The information is provided by the PM trainer.
K4b: The psychologist knows how to instruct and practice with the supporter.	P	**Individualized** [1] **instruction** [2,3] on how psychologists could practice with the supporter by providing a **demonstration** [1–3].	PM training	Psychologists are instructed by the PM trainer, who models the behavior (**appropriate model**). Opportunity to ask questions (**responding to needs**).
K5a: The supporter knows why it is important to help the client retrieve positive memories.	S	**Providing information** [1,2] about the expected outcome of this behavior.	Coaching by psychologist	The information is provided by the psychologist.
K5b: The supporter knows how to help the client retrieve positive memories.	S	**Individualized** [1] **instruction** [2,3] on how to help the client retrieve positive memories are provided by the psychologist, who also **models** [1–3] the behavior.	Coaching by psychologist	Psychologists (**appropriate model**) instruct the supporters and show the appropriate behavior through role play. Opportunity to ask questions (**responding to their needs**).
K6a: The psychologist and main nurse know why	P	**Providing information** [2] about the expected outcome of this behavior.	PM training	The information is provided by the PM trainer.

it is important to evaluate (and adjust) the pleasant activities plan together.	*N*	**Providing information** [1,2] about the expected outcome of this behavior.	*AiD training and e-learning*	The information is provided by the AiD trainer and in the e-learning.
K6b: The psychologist and main nurse know how to evaluate (and adjust) the pleasant activities plan together.	P	**Individualized** [1] **instruction** [2,3] and **demonstration** [1–3] of an evaluation.	PM training	Instruction and demonstration by the PM trainer (**appropriate model**); opportunity to ask questions (**responding to needs**).
	N	**Individualized** [1] **instruction** [2,3] and **demonstration** [1–3] of an evaluation.	*AiD training*	Instruction and demonstration by the AiD trainer (**appropriate model**); opportunity to ask questions (**responding to needs**).
K7a: The psychologist and main nurse know why it is important to implement PM.	P/*N*	**Providing information** [2] about the expected outcome of this behavior.	PM training/*AiD training and e-learning*	The information is provided by the PM/AiD trainer and in the e-learning.
K7b: The psychologist and main nurse how to implement PM.	P/*N*	**Individualized** [1] **instruction** [2,3] on how to implement PM.	PM training/*AiD training and e-learning*	Instruction by the PM/AiD trainer and in the e-learning (**appropriate** model). Opportunity to ask questions (**responding to needs**).
Self-efficacy (SE)				
SE1: The psychologist feels capable to report the positive memories.	P	Psychologists are **verbally persuaded** [1,3] about their capabilities.<br>**Guided practice** [1,2] with **feedback on performance** [2,3]**.**	PM training	Psychologists are persuaded by the PM trainer (**credible source**) why they are capable to report the memories. They practice and receive feedback on performance (**specific and individual**) from

				the PM trainer (**experienced person**) in the second PM training session.
SE2: The psychologist and main nurse feel capable to develop a detailed plan to improve positive contact with the client.	P	Psychologists are **verbally persuaded** [1,3] about their capabilities to develop a detailed plan together with nursing staff. **Guided practice** [1,2] with **feedback on performance** [2,3].	PM training	Psychologists are persuaded by the PM trainer (**credible source**) why they are capable to develop a detailed plan. They practice and receive feedback on performance (**specific and individual**) from the PM trainer (**experienced person**) in the second PM training session.
	N	Main nurses are **verbally persuaded** [1,3] about their capabilities to develop a detailed plan together with psychologists. **Guided practice** [1,2] with **feedback on performance** [2,3].	*AiD training, e-learning and coaching by psychologist*	Nurses are persuaded by the AiD trainer and in the e-learning (**credible sources**) why they are capable to develop a detailed plan. They practice and receive feedback on performance (**specific and individual**) by the psychologist through coaching.
SE3: The psychologist and main nurse feel capable to integrate the activity in the pleasant activities plan together.	P	Psychologists are **verbally persuaded** [1,3] about their capabilities.<br>**Guided practice** [1,2] with **feedback on performance** [2,3].	PM training	Psychologists are persuaded by the PM trainer (**credible source**) why they are capable to integrate the activity in the pleasant activities plan. They practice and receive feedback on performance (**specific and individual**) from the PM trainer (**experienced person**) in the second PM training session.

	N	Main nurses are **verbally persuaded** [1,3] about their capabilities. **Guided practice** [1,2] with **feedback on performance** [2,3].	*AiD training, e-learning and coaching by psychologist*	Nurses are persuaded by the AiD trainer and in the e-learning (**credible sources**) why they are capable to integrate the activity in the pleasant activities plan. They practice and receive feedback on performance (**specific and individual**) by the psychologist through coaching.
SE4: The psychologist feels capable to instruct and practice with the supporter.	P	Psychologists are **verbally persuaded** [1,3] about their capabilities.	PM training	Psychologists are persuaded by the PM trainer (**credible source**) why they are capable to instruct and practice with the supporter.
SE5: The supporter feels capable to help the client retrieve positive memories.	S	The supporter is **verbally persuaded** [1,3] about his/her capabilities, which are further strengthened by **guided practice** [1,2] with the psychologist and **feedback on performance** [2,3]. The supporter **plans coping responses** [1–3].	Coaching by psychologist	Supporters are persuaded by the psychologist (**credible source**) and practice with the psychologist through role play and receive feedback **(specific and individual)** from the psychologist (**experienced person**). Supporters learn to deal with potential problems (**identification of high-risk situations**).
SE6: The psychologist and main nurse believe they are capable to evaluate (and adjust) the	P	Psychologists are **verbally persuaded** [1,3] about their capabilities. **Guided practice** [1,2] with **feedback on performance** [2,3].	PM training	Psychologists are persuaded by the PM trainer (**credible source**) how they are capable to evaluate (and adjust) the pleasant activities plan. They practice and receive

pleasant activities plan together.				feedback **(specific and individual)** from the PM trainer (**experienced person**) in the second PM session.
	N	Main nurses are **verbally persuaded** [1,3] about their capabilities. **Guided practice** [1,2] with **feedback on performance** [2,3].	*AiD training, e-learning and coaching by psychologist*	Nurses are persuaded by the AiD trainer and in the e-learning (**credible sources**) why they are capable to evaluate (and adjust) the pleasant activities plan. They practice and receive feedback on performance (**specific and individual**) by the psychologist through coaching.
SE7: The psychologist and main nurse feel capable to implement PM.	P/*N*	Psychologists and main nurses are **verbally persuaded** [1,3] about their capabilities.	PM training/*AiD training and e-learning*	Psychologists and nurses are told by the PM trainer/AiD trainer and in the e-learning (**credible source**) why they are capable to implement PM.

AiD = Act in case of Depression; P = Psychologist; *N* = Main nurse; S = Supporter of PM, helps the client retrieve positive memories.

References

1. Kok, G.; Gottlieb, N.H.; Peters, G.J.Y.; Mullen, P.D.; Parcel, G.S.; Ruiter, R.A.C.; Fernández, M.E.; Markham, C.; Bartholomew, L.K. A taxonomy of behaviour change methods: An Intervention Mapping approach. *Health Psychol. Rev.* **2016**, *10*, 297–312.
2. Abraham, C.; Michie, S. A taxonomy of behavior change techniques used in interventions. *Health Psychol.* **2008**, *27*, 379–387.
3. Michie, S.; Wood, C.E.; Johnston, M.; Abraham, C.; Francis, J.J.; Hardeman, W. Behaviour change techniques: The development and evaluation of a taxonomic method for reporting and describing behaviour change interventions (a suite of five studies involving consensus methods, randomised controlled trials and analysis of qualitative data). *Health Technol. Assess.* **2015**, *19*, 1–187.
