# Supplementary material for: Applying Intervention Mapping to Improve the Applicability of Precious Memories, an Intervention for Depressive Symptoms in Nursing Home Residents
Source: Int J Environ Res Public Health. 2019 Dec 17;16(24):5163. doi: 10.3390/ijerph16245163 (PMC6950488; doi:10.3390/ijerph16245163)
Supplement: Supplementary file 1 [file ijerph-16-05163-s001.zip › Supplemental final/Table S1 Online questionnaire.pdf]

Table S1. Online questionnaire

Question	Answer options
Demographic information	
How old are you?	Numeric value
What is your gender?	Female/male
Which education have you received after high school?	Open question
Occupational information	
Which region do you work in?; What is your current profession?	Open question
How much working experience do you have with the target population of PM (in years)?	Numeric value
Reach	
We know that not all residents that might be indicated to receive a treatment actually receive that treatment. If the circumstances were ideal, how many residents do you estimate could have received PM, since you followed the PM training?; To how many residents did you propose PM since you followed the training?; With how many residents did you start PM since you followed the training?; With how many residents did you complete PM since you followed the training?	Numeric value
To which type of residents did you propose/apply PM?	Primarily somatic symptoms; cognitive symptoms; other: [own answer].
To which type of complaints did you propose/apply PM?	Depressive symptoms; anxiety symptoms; other: [own answer].
Relevance	
To what extent do you think PM can generally help to decrease depression in NH residents?; To what extent do you think PM can generally help to increase well-being in NH residents?; To what extent do you think PM meets the needs of NH residents?; To what extent do you think PM meets	10-point Likert scale from 1 = *not at all* to 10 = *very much*

the expectations of NH residents? To what extent are you satisfied with PM in general?; How satisfied are you with 'Session 1 - Introduction'?; How satisfied are you with 'Session 2 - Childhood'?; How satisfied are you with 'Session 3 - Adolescence'?; How satisfied are you with 'Session 4 - Adulthood'?; How satisfied are you with 'Session 5 – Closing'?	
Would you recommend the use of PM to other professionals?	Yes/No
Feasibility	
How feasible do you believe the design of PM is?; How feasible do you believe PM is in comparison to other treatments for depression in NH residents?; To what extent do you believe PM is appropriate for your work as a psychologist?; To what extent do you believe the general content of PM is applicable for the target group?; How applicable do you think 'Session 1 - Introduction' is?; How applicable do you think 'Session 2 - Childhood' is?; How applicable do you think 'Session 3 - Adolescence' is?; How applicable do you think 'Session 4 - Adulthood' is?; How applicable do you think 'Session 5 – Closing' is?; How easy do you think it is to execute PM?; To what extent do you feel competent to execute PM?	10-point Likert scale from 1 = *not at all* to 10 = *very much*
Barriers and facilitators	
Available time; the PM protocol; cooperation with my close colleagues; cooperation with colleagues from other disciplines; my supervisor/manager; changes within my team; changes within the organization; the culture within my organization; the extent to which PM is regarded important in my organization; information about PM from congresses, conferences, articles, etc.; other factors: [own answers].	10-point Likert scale from 1 = *very much obstructed implementation* to 10 = *very much facilitated implementation*
