# Supplementary figures and images for: Applying Intervention Mapping to Improve the Applicability of Precious Memories, an Intervention for Depressive Symptoms in Nursing Home Residents
Source: Int J Environ Res Public Health. 2019 Dec 17;16(24):5163. doi: 10.3390/ijerph16245163 (PMC6950488; doi:10.3390/ijerph16245163)

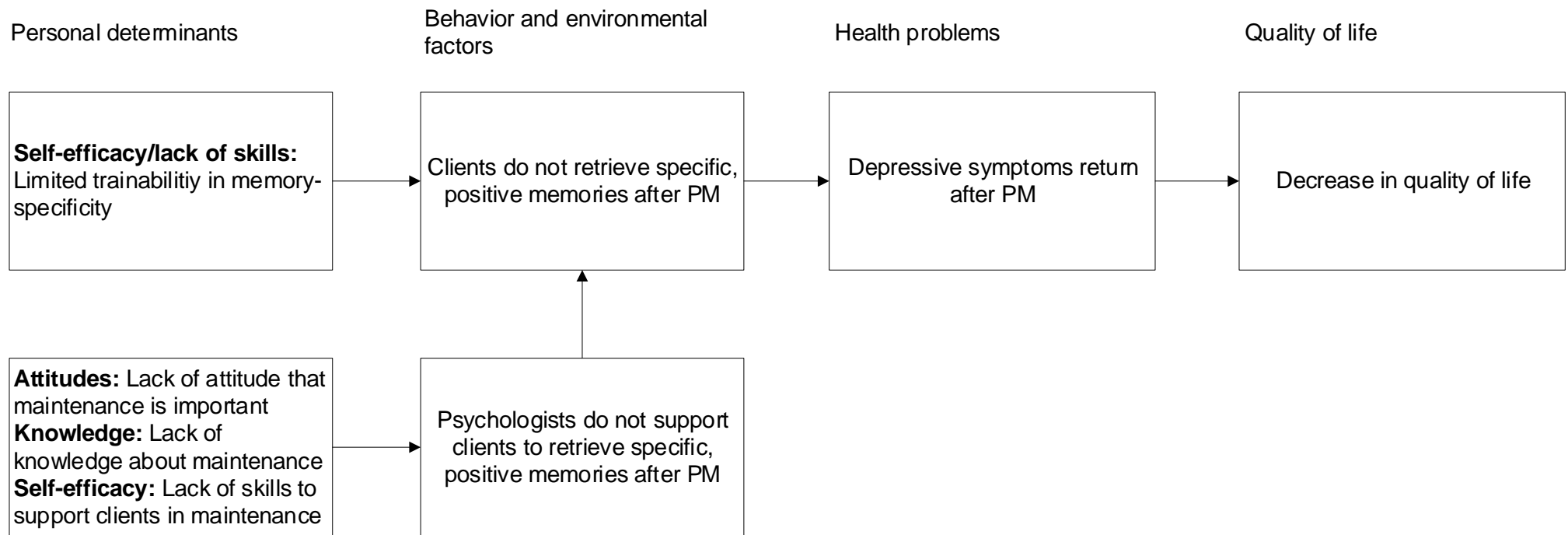

**Figure S1.** Logic model of the problem

Supplement: Supplementary file 1 [file ijerph-16-05163-s001.zip › Supplemental final/Figure S1 Logic model of the problem.pdf]
